# 5-fluorouracil modulated by leucovorin, methotrexate and mitomycin: highly effective, low-cost chemotherapy for advanced colorectal cancer

**DOI:** 10.1054/bjoc.2001.1732

**Published:** 2001-04

**Authors:** A Sobrero, A Guglielmi, M Cirillo, E Recaldin, G L Frassineti, C Aschele, A Ravaioli, P Testore, C Caroti, L Gallo, M A Pessi, E Cortesi, D Turci, F Grossi, R Labianca

**Affiliations:** GISCAD IOR and collaborating centres

**Keywords:** advanced colorectal cancer, biochemical modulation, 5-fluorouracil, mitomycin-C, schedule of administration

## Abstract

We have reported that an alternating regimen of bolus and continuous infusion 5-fluorouracil (FU) was superior to bolus FU in terms of response rate and progression-free survival in advanced colorectal cancer. Biochemical modulation was an essential part of this regimen and it was selective for the schedule of FU administration: bolus FU was in fact modulated by methotrexate (MTX) while continuous infusion FU was potentiated by 6-s-leucovorin (LV). Considering the low cost and the favourable report on the activity of mitomycin C (mito) added to CI FU, we have incorporated this agent in the infusional part of our treatment programme. 105 patients with untreated, advanced, measurable colorectal cancer were accrued from 13 Italian centres and treated with the following regimen. 2 biweekly cycles of FU bolus (600 mg/m^2^), modulated by MTX (24 h earlier, 200 mg/m^2^) were alternated with a 3-week continuous infusion of FU (200 mg/m^2^daily), modulated by LV (20 mg/m^2^weekly bolus). Mito, 7 mg/m^2^, was given on the first day of the infusional period. After a 1 week rest, the whole cycle (8 weeks) was repeated, if indicated. 5 complete and 34 partial responses were obtained (response rate, 37% on the intention to treat basis; 95% confidence limits, 28–46%). After a median follow-up time of 26 months, 37 patients are still alive. The median progression-free survival is 7.7 months with an overall survival of 18.8 months and a 2-year survival rate of 30%. The regimen was very well tolerated with fewer than 13% of patients experiencing WHO grade III–IV toxicity. These results are consistent with those obtained by our group in 3 previous trials of schedule specific biochemical modulation of FU. They also indicate a highly active, little toxic, inexpensive regimen of old drugs to be used (a) as an alternative to the more expensive combinations including CPT-11 or oxaliplatin or (b) as the basis for combination programmes with these agents. © 2001 Cancer Research Campaign http://www.bjcancer.com


*British Journal of Cancer* (2001) **84**(8), 1023–1028
© 2001 Cancer Research Campaign
doi: 10.1054/ bjoc.2001.1732, available online at http://www.idealibrary.com on IDEAL®

### 5-fluorouracil modulated by leucovorin, methotrexate and mitomycin: highly effective, low-cost chemotherapy for advanced colorectal cancer

**A Sobrero, A Guglielmi, M Cirillo, E Recaldin, GL Frassineti, C Aschele, A Ravaioli, P Testore, C Caroti, L Gallo, MA Pessi, E Cortesi, D Turci, F Grossi and R Labianca**

GISCAD, IOR and collaborating centres

**Summary** We have reported that an alternating regimen of bolus and continuous infusion 5-fluorouracil (FU) was superior to bolus FU in terms of response rate and progression-free survival in advanced colorectal cancer. Biochemical modulation was an essential part of this regimen and it was selective for the schedule of FU administration: bolus FU was in fact modulated by methotrexate (MTX) while continuous infusion FU was potentiated by 6-s-leucovorin (LV). Considering the low cost and the favourable report on the activity of mitomycin C (mito) added to CI FU, we have incorporated this agent in the infusional part of our treatment programme. 105 patients with untreated, advanced, measurable colorectal cancer were accrued from 13 Italian centres and treated with the following regimen. 2 biweekly cycles of FU bolus (600 mg/m$^2$), modulated by MTX (24 h earlier, 200 mg/m$^2$) were alternated with a 3-week continuous infusion of FU (200 mg/m$^2$ daily), modulated by LV (20 mg/m$^2$ weekly bolus). Mito, 7 mg/m$^2$, was given on the first day of the infusional period. After a 1 week rest, the whole cycle (8 weeks) was repeated, if indicated. 5 complete and 34 partial responses were obtained (response rate, 37% on the intention to treat basis; 95% confidence limits, 28–46%). After a median follow-up time of 26 months, 37 patients are still alive. The median progression-free survival is 7.7 months with an overall survival of 18.8 months and a 2-year survival rate of 30%. The regimen was very well tolerated with fewer than 13% of patients experiencing WHO grade III–IV toxicity. These results are consistent with those obtained by our group in 3 previous trials of schedule specific biochemical modulation of FU. They also indicate a highly active, little toxic, inexpensive regimen of old drugs to be used (a) as an alternative to the more expensive combinations including CPT-11 or oxaliplatin or (b) as the basis for combination programmes with these agents. © 2001 Cancer Research Campaign http://www.bjcancer.com

**Keywords**: advanced colorectal cancer; biochemical modulation; 5-fluorouracil; mitomycin-C; schedule of administration

A series of well designed, well conducted, drug company-sponsored randomized studies have first demonstrated the value of CPT-11 as second line treatment of patients with advanced colorectal cancer (CRC) (Cunningham et al, 1998; Rougier et al, 1998) and subsequently the value of CPT-11+ FU in the front line treatment of this disease (Douillard et al, 2000; Saltz et al, 2000). FDA has thus recently approved CPT-11 for this indication. Oxaliplatin is somewhat behind CPT-11, but it elicits similar optimism among oncologists, particularly in Europe (Maindrault et al, 1999; Giachetti et al, 2000).

The enthusiasm about these combinations and the pressure of drug companies is such that medical oncologists practising in the community, outside clinical trial settings, have a very hard time advising more conservative chemotherapy regimens to their patients. And the cost of treating this disease will increase dramatically in the next years.

While from a research prospective the small improvements afforded by the two new agents, particularly CPT-11, must be greeted as major breakthroughs, caution must be exercised from a broader perspective. The very small (<3 months) advantage in survival for CPT-11 + FU combination vs FU alone, must be weighed against the increased toxicity and cost of the combinations. Therefore on one side the ongoing research on how to best combine the different new agents must be enthusiastically supported, on the other, the search for alternative regimens of low cost and toxicity must not be discouraged.

In the mid 1990s, when biochemical modulation of FU was still dominating the scene of CRC treatment we developed the concept of schedule-specific biochemical modulation (Sobrero et al, 1997). We demonstrated that the fluoropyrimidine has different mechanisms of action depending on the dose schedule (Sobrero et al, 1993) and suggested that biochemical modulators should be specific for each schedule. A hybrid regimen alternating 2 biweekly cycles of sequential MTX → bolus FU, with a 3-week continuous infusion of FU+LV was tested in 2 phase II clinical studies (Sobrero et al, 1995; Aschele et al, 1998) and then demonstrated to be superior to modulated bolus FU in a recently published randomized trial (Sobrero et al, 2000). Based upon a British report (Ross et al, 1997) on the efficacy of mitomycin C added to CI FU, mito was added to the infusional part of our regimen in a phase II study that is the matter of this report.

*Received 7 August 2000*
*Revised 19 December 2000*
*Accepted 22 January 2001*

### MATERIALS AND METHODS

### Eligibility criteria

105 patients satisfying all of the following requirements were accrued into this 3 institution phase II trial between August 1997 and March 1999. (1) They had to have biopsy-proven relapsed or metastatic adenocarcinoma of the colon or rectum. There were 3 exceptions to the requirement for histologic confirmation of metastatic disease in patients with a history of resection for colorectal cancer no longer than 5 years before: (a) patients with 2 or more pulmonary nodules enlarging on serial chest X-rays and no other disease site accessible to biopsy. (b) Patients with 2 or more hepatic nodules and CEA >10 ng ml$^{-1}$ in at least 2 consecutive determinations; (c) patients with pelvic mass and new onset presacral pain. (2) The disease had to be measurable. Appropriate radiologic examinations (mostly CT scans) had to be obtained no longer than 1 month before the beginning of treatment to serve as a baseline for serial evaluation of the patient's disease status. (3) No prior chemotherapy for metastatic disease was allowed. Adjuvant chemotherapy was not an exclusion criterion provided that treatment was completed longer than 6 months before study entry. Radiation therapy was allowed as long as it did not encompass the indicator lesions. (4) ECOG performance status had to be ≤2. Serum bilirubin and creatinine levels were required to be less than 3.0 and 1.7, respectively, and aspartate and alanine aminotransferases less than 3 times the upper limits of normal. Granulocyte counts of greater than 1500/mm$^3$ and platelet counts of greater than 100 000/mm$^3$ were required.

Additional eligibility criteria included geographic accessibility, the absence of clinically relevant ascites and the absence of other medical conditions clearly contraindicating the delivery of any chemotherapy.

Staging should be performed within 1 month before study entry by means of clinical assessment, blood cell count, bilirubine and creatinine levels, transaminases, alkaline phosphatase, serum CEA, chest X-ray or thoracic CT scan, abdominal US, CT scan or NMR. Any imaging investigation that was abnormal due to malignant disease was repeated at 2-month intervals for the duration of treatment.

Informed consent was required. Before treatment, patients were informed as to: (a) the presence of metastatic colorectal cancer; (b) the poor prognosis of their disease; and (c) the experimental nature of this treatment protocol. Upon study entry all patients were given a schedule of drug treatment along with written information about the anticipated toxicities.

### Treatment plan

The backbone of the regimen consisted of the alternating regimen of bolus and infusional FU that we previously tested in a phase II trial and that was derived from 2 well studied regimens: sequential MTX and bolus FU (Marsh et al, 1991) and CI FU modulated by LV (Leichman et al, 1993). Mito was added to the infusional part. Figure 1 illustrates the regimen. One complete cycle of treatment consisted of 2 MTX → FU bolus treatments (200 mg/m$^2$ → 600 mg/m$^2$, respectively) given on days 1, 2 and 15, 16 along with LV rescue (15 mg) given p.o. q 6 hours × 6 doses, followed by 3 weeks of CI FU (200 mg/m$^2$) given from day 29 to day 49, modulated by weekly LV (20 mg/m$^2$). Mito, 7 mg/m$^2$, was administered once on the first day of the infusional period only. After one week of rest, the 2nd cycle was started on day 57, provided that the patient had recovered from toxicity. The entire duration of the cycle is thus 8 weeks. CI FU was administered through implanted catheters and a venus Port-a-cath (Pharmacia) connected to a portable programmable external pump (CADD-1, Pharmacia) or disposable elastomeres (Baxter). The infusional cassettes or elastomeres were changed weekly if no toxicity developed earlier.

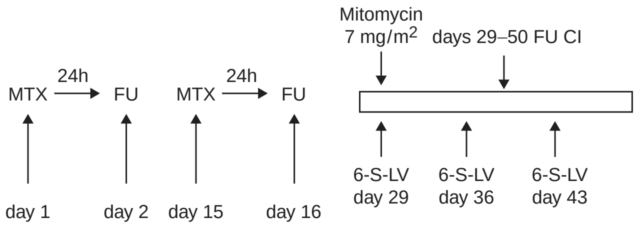

**Figure 1** Design of drug regimen. One cycle = 8 weeks. In the first part of the cycle, patients were given MTX 200 mg/m$^2$ i.v. diluted in 500 ml $D_5W$, infused in 1 h, day 1; FU 600 mg/m$^2$ i.v. bolus, day 2; (6S)LV, 15 mg p.o. every 6 h × 6, days 2–3, starting after FU bolus. In the second part of the cycle, patients were given FU, 200 mg/m$^2$/day CI × 3 weeks, and LV, 20 mg/m$^2$ i.v. bolus every week. Mito at 7 mg/m$^2$ was given on the first day of the infusional period

Toxicity was evaluated on days 15, 29, 36, 43, 50 and 57. Complete blood counts were obtained on the same days. Liver function tests, blood urea nitrogen, creatinine and electrolytes were obtained monthly.

Dose modification criteria for the MTX→FU regimen were as follows: no dose reduction for gastrointestinal grade I and II toxicity. For grade III diarrhoea or mucositis, the treatment was delayed until recovery and the doses of MTX and FU of the next cycle were decreased by 50%. The dose was reduced by 50% also for a WBC of <3000/mm$^3$ or platelets <75 000/mm$^3$ on the day of recycling. Treatment was discontinued in cases of grade IV toxicity.

CI FU was discontinued upon the first signs of mucositis and/or palmar-plantar dysaesthesia/burning, and resumed when these symptoms abated. In the case of severe (grade III) mucositis, the infusion was resumed at a reduced FU dose (50%). The dose of mito and that of LV during the infusional treatment were not modified in this study. Toxicity is expressed according to WHO criteria.

Due to the long duration of chemotherapy cycles (2 months), the response was evaluated after each cycle of treatment.

The duration of treatment depended on outcome. Upon documentation of CR, 2 additional cycles were given (4 additional months of treatment). In the case of a PR, treatment was continued until 2 consecutive CT scans, obtained 2 months apart, failed to demonstrate further tumour shrinkage. At that point, chemotherapy was stopped and the disease was monitored every 2 months. The same regimen was resumed upon documentation of tumour progression. In patients with disease stabilization, treatment was continued until evidence of progression was observed. No guidelines were given as regarded second line chemotherapy.

### Response evaluation

Patients who received at least 2 months of therapy (1 cycle) with adequate pretreatment and follow-up radiographic studies were considered assessable for response, as were patients who experienced rapid disease progression after at least 2 courses of bolus FU.

© 2001 Cancer Research Campaign

Measurable tumour was defined as a tumour mass that could be clearly measured in 2 dimensions by adequate imaging techniques.

A CR was defined as complete disappearence of all evidence of tumour and return of abnormal tests to normal levels for a minimum of 8 weeks. A PR was defined as a 50% or greater reduction in the sum of the products of the largest perpendicular diameters of all measured lesions, in the absence of progression of any lesion or the appearance of any new lesion for at least 8 weeks. Stable disease was defined as too small a change in measurable disease to meet the requirements for PR or progression, without the appearance of new lesions for a period of at least 8 weeks, provided that there was no worsening of symptoms. Disease progression was defined as the development of new areas of malignant disease, or an increase by at least 25% in the baseline area of the measured lesions in nonresponding patients, or a 25% increase in the size of measured lesions over that attained at best response. Indicator lesions were measured at each successive cycle. The baseline tumour areas and their variations at each successive evaluation were expressed in $cm^2$.

PFS and and overall survival were measured from the date of randomization to the date of disease progression as defined above, or to the date of death, respectively and calculated using the Kaplan–Meier method (Kaplan and Meier, 1958). Early progressions and deaths, toxic deaths if any, early withdrawals and deaths from other causes were included as failures.

The association between performance status and the proportion of responses were assessed using the Mantel test for trend (Mantel, 1963).

### Statistical methods

The general philosophy behind this phase II study was that mito should add some further activity to our alternating bolus-infusional modulated FU regimen. The new combination would be regarded as promising if an additional 10% response rate is added to our basic regimen. Since our standard alternating regimen without mito affords 30–35% RR, we were searching for a range of activity around 40 to 45% to consider the new combination for a new phase III study.

According to the two stage Simon's design, setting P0 = 30% and P1 = 45%, with an alfa error = 0.05 (reflecting the chances to accept an 'inactive' regimen) and a beta error = 0.1 (reflecting the chances of accepting as 'active' a truly inactive regimen), the treatment will be discontinued if less than 27 responses will be observed among the first 77 patients. Otherwise we will proceed to the second stage, where 33 responses over 88 patients will be necessary to define the study successful and proceed further with the clinical development of this combination.

### RESULTS

### Patients characteristics

Between August 1997 and March 1999, 105 patients meeting the eligibility criteria were registered from thirteen participating institutions.

Table 1 shows patient characteristics. 93% of patients had had surgery on the primary neoplasm. 20% patients had received prior adjuvant chemotherapy: 9 received FU+LV, 10 FU-levamisol and 2 FU-LV-levamisol. The median time between diagnosis of metastatic disease and study entry was 34 days. The wide range (3 to 273 days) of this figure is due to occasional patients with a very long natural history of their disease without treatment prior to study entry.

The minimum size of a measurable lesion was 1 cm × 1 cm. Lesions were measured by CT scan in 85 patients and ultrasound in 12 patients, the remaining being measured by CXR and NMR. Only 9 patients had lesions smaller than 2 cm × 2 cm and the median measured baseline tumour area was 22 $cm^2$ (range 1–194).

### Treatment outcome

6 patients were not evaluable for response. This was due to rapid clinical deterioration in 3 patients not allowing completion of the first cycle of treatment, 1 refusal to continue after the first drug administration, and lack of baseline tumour measurement in 2 patients. According to the intention-to-treat principle, all these patients were included in the analysis of response as failures, and in the PFS and survival analysis.

5 complete and 34 partial responses were obtained (response rate, 37% on the intention to treat basis; 95% confidence limits, 28–46%). In addition, a substantial percentage of patients (42%) had stable disease (Table 2). 22 failures were reported: 15 patients progressed after the first cycle of treatment, 3 patients showed a rapid disease progression before the end of the first cycle, 2 refused to continue treatment (1 just after the first drug

**Table 1** **Baseline patient characteristics**

| | |
|---|---|
| *N* | 105 |
| Median age (range) | 61 (39–77) |
| Male (%) | 60 |
| Female (%) | 40 |
| ECOG performance status, % | |
| 0 | 75 |
| 1 | 18 |
| 2 | 7 |
| Symptomatic (%) | 35 |
| Primary tumour site (%) | |
| Colon | 78 |
| Rectum | 27 |
| Number of organs involved (%) | |
| 1 | 71 |
| 2 | 26 |
| ≥3 | 4 |
| Site of metastases (%) | |
| Liver | 50 |
| Lung | 11 |
| Peritoneum/nodes | 10 |
| Prior adjuvant chemotherapy (%) | 21 |
| Median number of lesions measured per patient (range) | 3 (1–12) |
| Median baseline tumor area, $cm^2$ (range) | 22 (2–194) |
| Median baseline LDH (range) | 414 (90–1690) |
| Median baseline WBC × 1000 (range) | 7.3 (3.7–17.1) |
| Median baseline CEA level | 21 (1–3835) |

**Table 2** Response to treatment: intention to treat analysis

| | ***n* = 105** |
|---|---|
| Complete responses | 5 (5%) |
| Partial responses | 34 (32%) |
| Stable disease | 44 (42%) |
| Failures | 22 (21%) |
| Response rate (95% CL) | 37% (28–46) |

© 2001 Cancer Research Campaign 

administration, the other after the first cycle), and 2 had baseline tumour measurements missing. 90% of the cases of progression were due to the appearance of new lesions rather than enlargement of the indicator lesion.

The median time to achieve a partial or complete response was 61 (range, 49–246) days, with initial responses attained after one cycle (6 cases), 2 cycles (14 cases), 3 cycles (14 cases), 4 cycles (5 cases). The median number of cycles to obtain a CR was 3. Half of the responding patients showed continued tumour shrinkage and the median time to achieve the maximum clinical response was 132 (range 54–545) days. Among responding patients, the median maximum tumour mass reduction compared to the baseline measured tumour area was 74%, while among all 105 patients this value was 30%.

Patients obtaining a CR had a very low measured baseline tumour mass (median 3.2 $cm^2$, range 1.5 to 6.7). 4 out of 5 patients with complete response had liver disease only with multiple inoperable metastases (2, 3, 3 and 6 measured lesions, respectively); the other patient had 3 lung lesions as the only site of metastatic disease. None of these patients had received adjuvant chemotherapy and all had baseline CEA level < than the median value of the study population (21 ng $ml^{-1}$).

None of the patients on this study underwent surgical exploration in order to resect residual disease.

Only 8 of the 39 responses were obtained in patients with multiple metastatic sites, the rest being liver only (22 patients), lung only (5 patient) and extrahepatic intra-abdominal disease (4 patients). However the % RR in patients with 1 metastatic site ($n$ = 80) was similar to that observed in patients with 2 or more metastatic sites ($n$ = 25) (39% vs 32% $P$ = NS).

The baseline CEA level did not appear to influence the response rate (36% vs. 44%, in patients with CEA levels above or below 5 ng $ml^{-1}$; $P$ = NS).

The overall response rate was similar in patients with either colon (29 of 78 patients, 37%) or rectal primaries (10 of 27 patients, 37%).

Previous adjuvant treatment appeared to influence the clinical response but the difference did not reach statistical significance: 6 of 21 patients who had received adjuvant treatment responded (29% response rate) while 33 responses were observed among the 84 patients who had not received prior adjuvant chemotherapy (39% response rate).

The combined CR and PR rate was 41%, both in patients with an ECOG PS of 0 and 1. Symptomatic patients responded in 41% and asymptomatic in 40%.

Age and sex did not appear to influence the overall clinical response. 33% in < 60 vs 44 in > 60, $P$ = NS, male 36%, female 45%. 50% of symptomatic patients ($n$ = 42) improved their symptoms after the first cycle of treatment while only 13% got worse subjectively, the rest being stable.

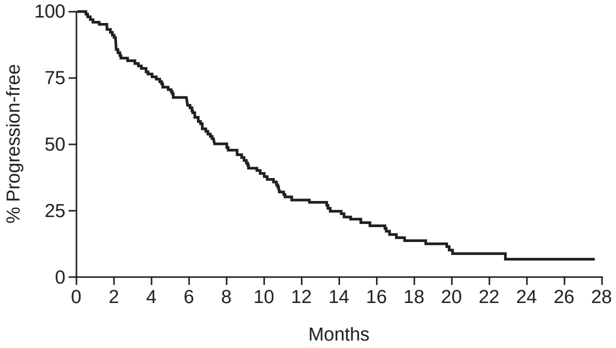

**Figure 2** Kaplan–Meier PFS curve for all 105 patients

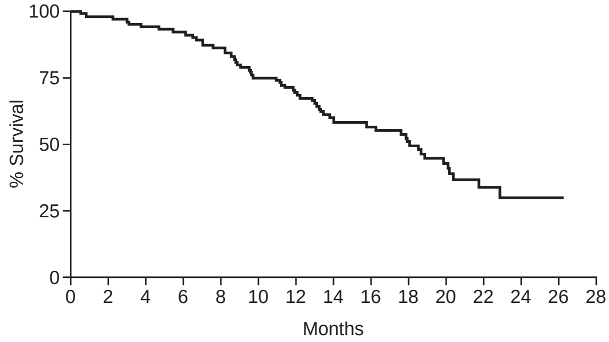

**Figure 3** Kaplan–Meier survival curve for all 105 patients

The median duration of response was 8.1 (range, 2–21) months and the median duration of stable disease was 6.4 (range, 1–20) months. All patients are now off treatment.

3 patients declined further chemotherapy while they were still responding; they were considered treatment failures as of the date the treatment was discontinued. After a median follow-up time of 26 months, 95 patients have progressed and 37 patients are still alive. The median progression-free survival was 7.7 months (Figure 2) with an overall survival of 18.8 months and a 2-year survival rate of 30% (Figure 3).

### Safety

273 cycles of treatment (2 months each) were administered, with a median of 3 cycles (range, 0–6) per patient.

Table 3 reports the worst toxicity of each type, suffered by each patient, across all cycles. The 2 parts of the regimen are considered separately. 538 cycles of sequential MTX→FU and 711 weeks of CI FU are the denominator of these percentages. No toxic deaths

**Table 3** Toxicity: worst WHO grade per patient across all cycles

| | MTX→FU toxicity grade (%) $n$ = 105 | | | | CIFU + 6-S-LV toxicity grade (%) $n$ = 103 | | | |
|---|---|---|---|---|---|---|---|---|
| **Toxicity** | **I** | **II** | **III** | **IV** | **I** | **II** | **III** | **IV** |
| Mucositis | 20 | 16 | 6 | 1 | 19 | 22 | 10 | 0 |
| Diarrhoea | 7 | 10 | 2 | 1 | 5 | 8 | 4 | 0 |
| Nausea/vomiting | 17 | 11 | 3 | 0 | 16 | 8 | 0 | 0 |
| Conjunctivitis | 11 | 2 | 0 | 0 | 18 | 4 | 0 | 0 |
| Hand-foot syndrome | 10 | 2 | 0 | 0 | 13 | 0 | 1 | 0 |

© 2001 Cancer Research Campaign were reported following either part of the regimen. Stomatitis was the most commonly observed severe side effect in both parts: fewer than 7% of patients in the bolus part of the programme and 10% in the CI part. Grade IV toxicity was not reported during the infusional part of the regimen.

No prophylactic antiemetics were used during CI FU, while either metoclopramide or metoclopramide plus steroids were used during the first 3 days of the bolus schedule.

Only 5 patients (5%) had catheter-related complications requiring admission to hospital: 1 with sepsis and 3 with thrombosis. In addition, 1 patient had the treatment changed following removal of a damaged catheter.

The cost of 3 cycles (median duration of treatment) of this chemotherapy (6 months) in the Italian setting was calculated assuming that no complication occurred, that the treatment was done on an outpatient basis and that 7 accesses/cycle were necessary. Such cost can be estimated to be $11 400 US to which an additional 1500 USD for the catheter and its implantation. This may be compared to the recently reported regimen of FU + CPT-11 where the cost for the same length of treatment (12 biweekly administrations = 6 months) adds up to $31 500 US plus the same cost for catheter implantation, not counting the cost for the management of the severe toxicities occurring in up to 36% and 46% of patients for diarrhoea and leukopenia, respectively (Douillard et al, 2000).

### DISCUSSION

The clinical research on CRC is now fully dominated by CPT-11, oxaliplatin and the oral fluoropyrimidines. In addition, the novel agents such as C-225 (Waksal, 1999), SU 5416 (Rosen et al, 2000), RhuMabVEGF (Bergsland et al, 2000) and ONYX 0–15 (Reid et al, 2000) show their first signs of clinical activity in early phase II studies in this disease. Scheduling of FU, biochemical modulation and mito therefore are certainly not ‘cutting edge’ clinical research. Nevertheless, the present trial started 3 years ago when the above ‘booming’ prospectives were still far away, raltitrexed was the new big thing around, the novel agents were confined to the preclinical world and our review on the importance of FU scheduling was just published on a leading oncology journal (Sobrero et al, 1997). The still very short life span of the results obtained with CPT-11 and oxaliplatin combinations would suggest to be more cautious, especially considering cost and toxicity.

In the last 3 years we have published 3 clinical trials based upon our hypothesis that FU is indeed 2 different drugs depending upon the schedule of administration (Sobrero et al, 1995, 2000; Aschele et al, 1998). If that hypothesis holds up in the clinic, maximal enhancement of bolus FU is more likely obtained with drugs that enhance the RNA effect of the fluoropyrimidine such as MTX, while LV, that selectively enhances the TS inhibitory activity of FU, may result in greater potentiation when the fluoropyrimidine is administered as continuous infusion. In each of the 3 studies done with this regimen, the response rate was always greater than 35%, the PFS and OS always longer than 6.5 and 15 months respectively. When a British group (Ross et al, 1997) reported that CI FU + mito was superior to CI FU alone in terms of response rate and PFS (54% response rate, 7.9 months in PFS and 14 months in OS were obtained in the combination arm), it seemed logical to us to incorporate that low-cost drug into the infusional part of our regimen. The response rate in our trial was not particularly high, but the PFS and OS were the longest observed among our multicentric studies on a total of more than 300 patients with similar characteristics.

It is obviously unfair to compare our phase II data with those of the randomized British trial (the shorter OS in the British trial is certainly explained by the randomized nature of their study), however both studies suggest a renewed role for an old, cheap and non-toxic (at these doses) agent in the treatment of this disease.

The consistency of the results we have obtained throughout our 4 trials in this field makes us consider the results of this latest phase II very encouraging, nevertheless we cannot propose this regimen as standard first-line treatment because of the lack of phase III data.

Since toxicity affords, the logical next step is to incorporate the new cytotoxics into this old style, but still very effective regimen. Our ongoing trial in fact investigates the addition of oxaliplatin to MTX $\rightarrow$ FU bolus, leaving the infusional part unchanged. If this regimen shows a substantial improvement in activity with tolerable toxicity (say an additional 15–20% response rate, and a concomitant further prolongation of PFS by a couple of months), we will randomize our new regimen vs whatever combination will be regarded as standard at that time. Should the benefit afforded by the addition of oxaliplatin be more limited, we will instead proceed to a new phase II incorporating CPT-11 into our schedule specific regimen.

### ACKNOWLEDGEMENT

The following investigtors have contributed to patient accrual: P Foa, Ospedale S Paolo, Milano; S Barni, Ospedale Treviglio; E Piazza, Ospedale Sacco, Milano; G Martignoni, Ospedale S Carlo, Milano. We are grateful to Mrs Carla Curti for data management and secretarial support. Supported by grants Associazione Italiana Ricerca Cancro, Milano and CNR Biotechnology and Oncology Strategic Project 2000.

### REFERENCES

Aschele C, Guglielmi A, Frassineti GL, Milandri C, Amadori D, Labianca R, Vinci M, Tixi L, Caroti C, Ciferri E, Verdi E, Rosso R and Sobrero A (1998) Schedule selective biochemical modulation of 5-fluorouracil in advanced colorectal cancer: a multicentric phase II study. *British J Cancer* **77**: 341–346

Bergsland E, Hurwitz H, Fehrenbacher L, Meropol MJ, Novotny WF, Gaudreault J, Lieberman G, Kabbinavar F (2000) A randomized phase II trial comparing rhuMAb VEGF (recombinant humanized monoclonal antibody to vascular endothelial cell growth factor) plus 5-fluorouracil/leucovorin (FU/LV) to FU/LV alone in patients with metastatic colorectal cancer. *Proc Am Soc Clin Oncol* **19**: 936

Cunningham D, Pyrrhonen S, James RD, Punt CJA, Hickih TF, Heikkila R, Johannesen TB, Starkhammar H, Topham CA, Awad L, Jacques C and Herait P (1998) Randomised trial of irinotecan plus supportive care versus supportive care alone after fluorouracil failure for patients with metastatic colorectal cancer. *Lancet* **352**: 1413–1418

Douillard JY, Cunningham D, Roth AD, Navarro M, James RD, Karasek P, Jandik P, Iveson T, Carmichael J, Alakl M, Gruia G, Awad L and Rougier P (2000) Irinotecan combined with fluorouracil alone as first-line treatment for metastatic colorectal cancer: a multicentre randomised trial. *Lancet* **355**: 1041–1047

Giachetti S, Perpoint B, Zidani R, Le Bail N, Faggiuolo R, Focan C, Chollet P, Llory JF, Letourneau Y, Coudert B, Bertheaut-Cvitkovic F, Larregain-Fournier D, Le Rol A, Walter S, Adam R, Misset JL, Lévi F (2000) Phase III multicenter randomized trial of oxaliplatin added to chronomodulated fluorouracil-leucovorin as first line treatment of metastatic colorectal cancer. *J Clin Oncol* **18**: 136–147

Kaplan E and Meier P (1958) Non-parametric estimation from incomplete observations. *J Am Stat Assoc* **53**: 457–458

*© 2001 Cancer Research Campaign* 

Leichman CG, Leichman L and Spears CP (1993) Prolonged infusion of fluorouracil with weekly bolus leucovorin: a phase II study in patients with disseminated colorectal cancer. *J Natl Cancer Inst* **85**: 41–44

Maindrault-Goebel F, Louvet C, Andre T, Carola E, Lotz JP, Molitor JL, Garcia ML, Gilles-Amar V, Izrael V, Krulik M, de Gramont A (1999) Oxaliplatin added to the simplified bimonthly leucovorin and 5-fluorouracil regimen as second-line therapy for metastatic colorectal cancer (FOLFOX6). GERCOR *Eur J Cancer* **35**: 1338–1342

Mantel M (1963) Chi-square tests with one degree of freedom: extensions of the Mantel-Haenszel procedure. J Am Stat Assoc **58**: 690–700

Marsh JC, Bertino JR, Katz KH, Davis CA, Durivage HJ, Rome LS, Richards IIF, Capizzi RL, Farber LR, Pasquale DN, Stuart R, Koletsky AJ, Makuch R and O'Hollaren K (1991) The influence of drug interval on the effect of methotrexate and fluorouracil in the treatment of advanced colorectal cancer. J Clin Oncol **9**: 371–380

Reid T, Galanis E, Abbruzzese J, Sze D, Romel L, Hatfield M, Rubin J, Kirn D (2000) Hepatic artery infusion of onyx-015, a selectively-replicanting adenovirus in combination with 5-FU/leucovorin for gastrointestinal carcinoma metastatic to the liver. *Proc Am Soc Clin Oncol* **19**: 953

Rosen PJ, Amado R, Hecht JR, Chang D, Mulay M, Parson M, Laxa B, Brown J, Cropp G, Hannah A, Rosen L (2000) A phase I/II study of SU5416 in combination with 5-FU/leucovorin in patients with metastatic colorectal cancer. *Proc Am Soc Clin Oncol* **19**: 5D

Ross P, Norman A, Cunningham D, Webb A, Iveson T, Padhani A, Prendiville J, Watson M, Massey A, Popescu R and Oates J (1997) A prospective randomized trial of protracted venous infusion fluorouracil with or without mitomycin C in advanced colorectal cancer. *Annals Oncol* **8**: 995–1001

Rougier P, Van Custem E, Bajetta E, Niederle N, Possinger K, Labianca R, Navarro M, Morant R, Bleiberg H, Wils J, Awad L, Hérait P and Jacques C (1998) Randomised trial of irinotecan versus fluorouracil by continuous infusion after fluorouracil failure in patients with metastatic colorectal cancer. *Lancet* **352**: 1407–1412

Saltz JB, Douillard J, Pirotta N, Awad L, Elfring GL, Gruia G, Locker PK, Alakl M, Knight RD and Miller LL (2000) Combined analysis of two phase III randomized trials comparing irinotecan, fluorouracil, leucovorin vs fluororuacil alone as first-line therapy of previously untreated metastatic colorectal cancer. *Proc Am Soc Clin Oncol* **19**: 938

Sobrero A, Aschele C, Guglielmi A, Mori AM, Melioli GG, Rosso R and Bertino JR (1993) Synergism and lack of cross-resistance between short-term and continuous exposure to fluorouracil in human colon adenocarcinoma cells. *J Natl Cancer Inst* **85**: 1937–1944

Sobrero A, Aschele C, Guglielmi A, Mori AM, Tixi L, Bolli E, Rosso R, Mammoliti S, Bertoglio S, Rollandi G, Bruzzi P and Bertino JR (1995) Schedule-selective biochemical modulation of 5-fluorouracil: a phase II study in advanced colorectal cancer. *Clin Cancer Res* **1**: 955–960

Sobrero AF, Aschele C and Bertino JR (1997) Fluorouracil in colorectal cancer: a tale of two drugs. Implications for biochemical modulation. *J Clin Oncol* **15**: 368–381

Sobrero A, Zaniboni A, Frassineti GL, Aschele C, Guglielmi A, Giuliani R, Ravaioli A, Lanfranco C, Caroti C, Arnoldi E, Barni S, Gallo L, Pessi MA, Turci D, Cortesi E, Grossi F, Frontini L, Piazza E, Bruzzi P and Labianca R (2000) Schedule specific biochemical modulation of 5-fluorouracil in advanced colorectal cancer: a randomized study. *Annals of Oncol* **11**: 1–8

Waksal HW (1999) Role of an anti-epidermal growth factor receptor in treating cancer. *Cancer Metastasis Rev* **18**: 427–436

 *© 2001 Cancer Research Campaign*
